# *COQ8A*-Ataxia as a Manifestation of Primary Coenzyme Q Deficiency

**DOI:** 10.3390/metabo12100955

**Published:** 2022-10-08

**Authors:** Justyna Paprocka, Magdalena Nowak, Piotr Chuchra, Robert Śmigiel

**Affiliations:** 1 Department of Pediatric Neurology, Faculty of Medical Sciences in Katowice, Medical University of Silesia, 40-752 Katowice, Poland; 2 Students’ Scientific Society, Department of Pediatric Neurology, Faculty of Medical Sciences in Katowice, Medical University of Silesia, 40-752 Katowice, Poland; 3 Department of Family and Pediatric Nursing, Wroclaw Medical University, 51-618 Wrocław, Poland

**Keywords:** primary coenzyme Q10 deficiency-4, *COQ8A*-ataxia, coenzyme Q10, cerebellar ataxia, COQ8A, primary coenzyme Q10 deficiency

## Abstract

*COQ8A*-ataxia is a mitochondrial disease in which a defect in coenzyme Q10 synthesis leads to dysfunction of the respiratory chain. The disease is usually present as childhood-onset progressive ataxia with developmental regression and cerebellar atrophy. However, due to variable phenotype, it may be hard to distinguish from other mitochondrial diseases and a wide spectrum of childhood-onset cerebellar ataxia. *COQ8A*-ataxia is a potentially treatable condition with the supplementation of coenzyme Q10 as a main therapy; however, even 50% may not respond to the treatment. In this study we review the clinical manifestation and management of *COQ8A*-ataxia, focusing on current knowledge of coenzyme Q10 supplementation and approach to further therapies. Moreover, the case of a 22-month-old girl with cerebellar ataxia and developmental regression will be presented.

## 1. Introduction

Ataxia may present as the first and predominant symptom of many neurological diseases in the pediatric population. The overall prevalence of ataxia is 26 cases per 100,000 children [1]. Genetic background is the most common cause of ataxia in children. Different etiologies are infections, immunological disorders, congenital defects, and metabolic disturbances [1].

Rapid development and better accessibility to next generation sequencing (NGS)have contributed to the discovery of many new variants of genetic diseases and have enabled more accurate molecular diagnosis, especially in the group of patients with autosomal recessive cerebellar ataxia (ARCA) [2]. ARCA may be particularly useful in the diagnosis of patients with overlapping clinical phenotypes and heterogeneous genetic background, which are characteristic of these conditions [1]. One of the examples of ARCA is primary coenzyme Q10 deficiency-4 (COQ10D4) caused by a mutation in the *COQ8A* gene [2].

Primary coenzyme Q10 deficiency-4 (also known as *COQ8A*-ataxia, autosomal recessive cerebellar ataxia 2 (ARCA2), or autosomal recessive spinocerebellar ataxia-9 (SCAR9)) is a mitochondrial disorder caused by the mutation in the *COQ8A* gene (previously: *ADCK3*, *CABC1*) and characterized by a reduced level of coenzyme Q10 (CoQ10, ubiquinone) [3]. This condition belongs to the group of primary coenzyme Q10 deficiency disorders containing five major phenotypes: encephalomyopathy, cerebellar ataxia, infantile multisystemic form, nephropathy, and isolated myopathy [4]. Heterogeneous phenotypes of the disease are caused by a biallelic pathogenic variant of one of the nine genes involved in the biosynthesis of coenzyme Q10: *COQ2*, *COQ4*, *COQ6*, *COQ7*, *COQ8A*, *COQ8B*, *COQ9*, *PDSS1,* and *PDSS2* genes [3]. Cerebellar ataxia is the most common feature of this condition [4]. The prevalence of *COQ8A*-ataxia is unknown; so far, 123 patients from 24 different countries have been described [2,5,6,7,8,9,10,11,12,13,14,15,16,17,18,19,20,21,22,23,24,25,26,27,28,29,30,31,32,33,34,35,36,37]. In this group, only one patient from Poland has been reported [31].

In this article, we report a case of a 22-month-old girl with ataxia and development regression, the second case of COQ10D4 in Poland described so far. Moreover, we provide a literature review of the features and management of coenzyme Q10 deficiency-4, focusing on aspects of CoQ10 therapy and further perspectives of alternative therapy for this condition. Differential diagnosis of other mitochondrial diseases presenting with ataxia is also depicted.

## 2. Case Description

A 22-month-old girl was admitted to the Department of Pediatric Neurology for a diagnosis of developmental regression. The girl was born at 40 weeks of gestational age, after an uncomplicated pregnancy. Apgar scores were 10. The newborn’s birth weight was 3450 g, head circumference was 34 cm, and body length was 53 cm. On neonatal examination, clinodactyly of the 5th fingers was detected. Family history was unremarkable.

According to the mother’s description, the patient’s psychomotor development was normal up to the age of 10 months. The girl achieved the following developmental milestones: babbling at the age of 4 months and getting to a sitting position by herself at the age of 6 months. At the age of 10 months, the girl started crawling, getting up with support and saying single words. Then, after the febrile infection, the parents observed inhibition and regression of acquired milestones and also difficulty with maintaining balance. At the age of 22 months, the girl was not able to get up on her own, point her finger, or execute commands. She did not speak. Moreover, episodes of gazing with the loss of contact with the child were reported.

Physical examination at the age of 22 months revealed limited eyeball elevation with no signs of the dysfunctions of other cranial nerves, as well as reduced muscle tension and positional asymmetry with shortening of the left side. No other abnormalities were observed in the ophthalmological evaluation. The auditory brainstem response (ABR) test was normal. The results of laboratory tests revealed elevated levels of serum lactate, CK, CK-MB, LDH, and liver function parameters. Other results (morphology, urinalysis, capillary gases, ions) were within the normal range. No abnormalities were detected in the abdominal ultrasound examination, and no echocardiographic sign of congenital heart defect was visible. Brain and cervical spine MRI showed images adequate for the patient’s age. The sleep EEG examination revealed an abnormal tracing with periodically fragmented 0.5–3 s series of generalized slow waves (mainly theta), sometimes with accompanying sharp waves of the highest amplitude up to 400–500 μV. No seizure activity was found during video monitoring. Psychiatric evaluation allowed the exclusion of autism spectrum disorders and confirmed the developmental delay. A rehabilitation treatment program was provided.

To exclude Pompe disease and Niemann-Pick disease type C, adequate genetic tests were performed. Suspecting the neurometabolic background of the disease, the urine organic acids by GC/MS and the acylcarnitine profile (tandem mass spectrometry) as well as amino acids profile in serum were ordered. The urine organic acid test revealed excretion of 3-hydroxyglutaric acid, α-ketoglutaric acid, and other citric acid cycle intermediates as well as excretion of lactic acid, pyruvic acid, and hippuric acid. In the acylcarnitine profile test, slightly elevated quantities of 3-hydroxydodecanoyl carnitine were detected.

In the physical examination at the age of 2.5 years, the patient sat alone with symptoms of ataxia. She was able to stand up independently but had trouble maintaining balance, did not speak, and made inarticulate sounds. Moreover, no eyeball elevation with poorly expressed ptosis and hypotonia were found. No signs of nystagmus, strabismus, or intentional tremor were observed.

The blood test showed constantly elevated levels of aminotransferases (AST—118 U/L, ALT—68 U/L), creatine kinase (752 U/L), creatine kinase MB (96.9 U/L), lactate dehydrogenase (667 U/L), lactic acid (2.41 mmol/L), and pyruvic acid (1.4 mg/dL).The lactic acid test (ischemia test, performed with an inflated blood pressure cuff) showed elevation of the lactic acid level in 30 min and normalization in 90 and 120 min. Immunological tests for EBV, CMV, HBV, and HCV infections were negative. The cerebrospinal fluid analysis revealed increased levels of lactic acid (30.6 mg/dL). Diagnostics were extended to plasma and cerebrospinal fluid amino acids tests; the test results were within the normal range. Biogenic amine testing was also conducted, which allowed the exclusion of neurotransmission disorders. The patient scored five points on the Nijmegen scale, and due to the suspicion of mitochondrial disease, molecular testing was ordered [38]. The control MRI examination did not reveal any signs of cerebellar atrophy.

Whole exome analysis (WES) with mitochondrial genome analysis and analysis of known pathogenic variants lying outside the protein-coding regions, described in the ClinVar database, were performed. Twist Human Core Exome, Twist mtDNA Panel, Twist RefSeq Panel, and ClinVar Custom Panel (Twist Bioscience) kits were used. The presence of the molecular biallelic pathogenic variant c.811C>T(p.Arg271Cys) of the *COQ8A* gene was detected. The patient’s parents are asymptomatic carriers of this variant. The studied variant of the *COQ8A* gene has been described in patients with ubiquinone deficiency (primary CoQ10 deficiency) with cerebellar ataxia.

The patient was administered coenzyme Q10 (300 mg/day).Three months after the beginning of the therapy, the parents observed improvement in communications with their child and better growth. However, improvement in movement development and ataxia were not observed. The patient is under the constant care of a neurologist, cardiologist, audiologist, and rehabilitation specialist.

## 3. Primary Coenzyme Q10 Deficiency-4

### 3.1. Molecular Background

**Figure 1:**
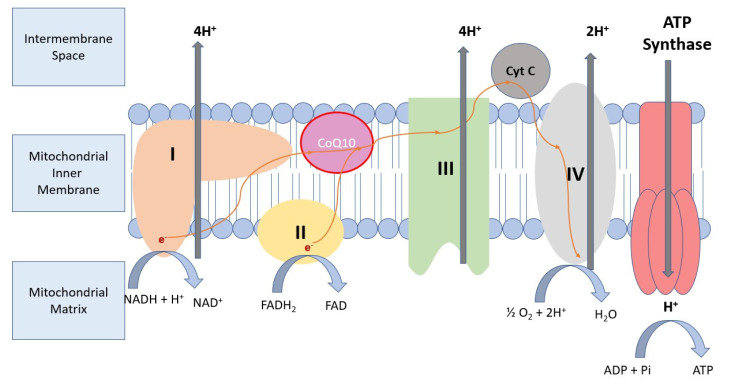
Role of CoQ10 in the mitochondrial electron transport chain. CoQ10—lipid-soluble component of the mitochondrial inner membrane, which acts as an electron acceptor at the level of the mitochondrial respiratory chain. CoQ10 carries electrons from complex I and complex II to complex III, contributing to ATP production. ADP: Adenosine diphosphate; CoQ10: Coenzyme Q10;Cyt C: Cytochrome C; e^−^: electron; FAD: flavin adenine dinucleotide; FADH: reduced flavin adenine dinucleotide; H+: proton; Pi: inorganic phosphate.

Coenzyme Q10 is an integral part of the mitochondrial electron transport chain, which generates a protomotoric force used to synthesize adenosine triphosphate. The main role of CoQ in the electron transport chain is the temporary storage and transport of electrons between proteins (Figure 1). CoQ is a lipophilic antioxidant and takes part in membrane structure maintenance [39,40]. Mutation in genes participating in CoQ biosynthesis results in humans in the spectrum of primary coenzyme Q10 deficiency disorders. The most frequent form of these conditions is COQ10D4, which is caused by *COQ8A* mutation [39]. *COQ8A* is part of the UbiB family, which consists of 25% microbial protein kinase-like (PKL) genes [41]. It is localized in the matrix face of the inner mitochondrial membrane and interacts with the CoQ biosynthesis enzymes. COQ8A acts as a specific stabilizer of a CoQ biosynthesis complex through unorthodox functions of PKL. These unorthodox functions include ATPase activity that enhances complex Q interactions or supports complex Q assembly, specific lipid bindings and small molecule kinase activity that phosphorylates a complex Q component, compared to the canonical Ser/Thr/Tyr protein kinase model. In mouse models of *COQ8A*-ataxia, pathogenic variants lead to progressive cerebellar ataxia associated with the dysfunction of Purkinje cells [39,40].

COQ10D4 is inherited in an autosomal recessive manner [5]. Mollet et al. first described homozygosity or compound heterozygosity for mutations in the *COQ8A* gene in patients presenting with cerebellar ataxia [5]. In the largest cohort of patients with COQ10D4, 44 different likely pathogenic variants of the *COQ8A* gene were found [2]. The most frequent subgroup of *COQ8A* mutation consists of missense variants [2,11]. Nonsense mutations, splicing, deletions, insertions and duplications, and complex rearrangement mutations were also reported [11]. Mutations are localized almost across the whole *COQ8A* gene; however, many missense variants were found to be clustered near or within functional motifs like mitochondrial target sequence (MTS), transmembrane domain (TM),KxGQ motif, AAAS motif of A-rich loop, ExD motif, catalytic loop, and Mg-binding DFG motif of the activation loop [2]. Frameshift and stop variants cause the termination of the COQ8A protein, when missense mutation results in various changes in *COQ8A* structure, leading to steric and/or electrostatic clashes, transmembrane or GQα helix disruption, loss of amino acid interactions, and mitochondrial targeting impairments [2].

Traschutz et al. revealed the phenotype-genotype correlation in patients with *COQ8A*-ataxia. According to this study, patients with biallelic loss of function (LOF)variants presented more often with phenotypes limited to only cerebellar ataxia symptoms (“ataxia-simplex phenotype”), compared to missense variants. Consequently, missense variants were associated with a multisystemic spectrum of symptoms, including cognitive impairment, epilepsy, myoclonus, dystonia, or myopathic features [2]. However, in patients with biallelic missense variants within the AAAS motif, the ataxia-simplex phenotype and the absence of seizure disorder were often found. Moreover, variants clustered at the KxGQ domain characterized the occurrence of developmental delay, epilepsy, and pyramidal signs [2].

### 3.2. Clinical Manifestations

Patients affected with COQ10D4 disease present a diverse spectrum of symptoms [2]. Extremely variable clinical manifestation has been reported even in siblings [18,29]. It has been shown that the onset of the disease appears in 50% of patients before 6 years of life, and most of the patients develop obvious symptoms before the age of 15 years [2,32]. However, in the older reported patients, the first symptoms of the disease occur at the age of 45 years [2,27].

According to the largest described cohort of patients with COQ10D4 disease, the most frequent symptom of the disease is cerebellar ataxia, which occurs in all patients [2,32]. The onset of ataxia ranges from 18 months to 15 years; however, adult-onset ataxia has been reported [2,27]. In the group of pediatric patients, gait disturbances (unsteady gait) were the most frequently reported complaint. Common manifestations of cerebellar dysfunction are hand clumsiness and dysarthric speech. Neurological examination may reveal dysmetria, dysdiadochokinesia, intention tremor, and variable range of hypotonia [32,33,35]. Apart from cerebellar ataxia, COQ10D4 is also characterized by the spectrum of other movement disturbances, especially hyperkinetic movement disorders, which may manifest as myoclonus, dystonia, and head/postural tremors or chorea [31,32]. It should be highlighted that the onset of the disease with only hyperkinetic features has been described [2]. Eye movement disorders like a saccadic ocular pursuit, gaze palsy, ophthalmoplegia, or oculomotor apraxia may be presented. Moreover, ptosis, nystagmus, and esotropia extend the ophthalmological spectrum of symptoms [2,4,11].

Another significant manifestation of *COQ8A*-ataxia is cognitive impairment. Cognitive impairment may occur in approximately 49% of patients and may be accompanied by a spectrum of intellectual disabilities [2]. In younger children, a developmental delay may occur as a first symptom. A common feature of mitochondrial diseases like *COQ8A*-ataxia is regression of development in patients who previously achieved milestones at the proper age [14]. Adolescents and older patients may require psychiatric care to treat distinctive disorders (depression, anxiety, psychotic disorder, or behavioral disturbances) in this particular group of patients [2,18].

Other neurological features that usually appear in juvenile age are epilepsy, which may affect about 20% of patients with COQ10D4. Generalized tonic-clonic seizures are the most often reported. The wide range of seizure disorders includes well-controlled to multidrug-resistant epilepsy and even cases of epilepsia partialis continua and status epilepticus [12,14].The clinical presentation, also characteristic of juvenile age, is myopathic disorder, typically presented as muscle weakness or exercise intolerance [14,33]. In the group of less frequently reported neurological symptoms, pyramidal signs, disturbances in vibration sense, and swallowing difficulties have been described [11,27,32].

At the onset, the condition is usually presented as gait ataxia and loss of balance, and then with the progression of the disease, other cerebellar signs, including limb incoordination, abnormal eye movements, tremor, and dysarthria appear [27,32]. However, due to the complexity of the neurological features involved in the clinical presentation of *COQ8A* mutations, COQ10D4 should be considered in the differential diagnosis of unexplained, complex neurological symptoms, even when cerebellar ataxia is not a major feature [17].

It should be emphasized that symptoms typically observed in patients with mitochondrial diseases, such as hearing loss, diabetes, cataract, and optic nerve atrophy, are rarely observed. As well, features such as cardiomyopathy, pigmentary retinopathy, and renal or liver dysfunction are non-typically reported in *COQ8A*-ataxia cases [2].

### 3.3. Diagnosis

Similar to other mitochondrial disorders, in the whole group of patients with primary coenzyme Q10 deficiency, initial laboratory investigation may reveal a high concentration of serum or plasma lactate [42]. However, according to the literature, elevated levels of serum lactate may not be present in the majority of patients with COQ10D4; however, this may be the first laboratory finding, especially in patients presenting with muscle involvement. It has been also described that in some cases serum lactate levels may be normal at rest and elevated only after aerobic exercise [4,7,13,32]. Even though CSF lactate concentration is found to be a more specific parameter, especially when the pathological process involves CNS, it may present within the normal range in COQ10D4 patients [32,42]. Moreover, metabolic evaluation, including urine organic acid profile, and concentration of serum amino acids are unremarkable in most cases [11,14,18,35]. In some patients, an increased level of phosphocreatine kinase (CK), creatine kinase-myocardial isoenzyme (CK-MB), lactate dehydrogenase (LDH), or liver parameters has been described [34,35].

Brain imaging is an integral part of the diagnosis of ataxia in children. It has been shown that mitochondrial diseases are the most frequent cause of cerebellar atrophy in the group of patients with childhood-onset ataxias [43]. Cerebellar atrophy is a major finding in MRI in patients with *COQ8A*-ataxia, which appears in almost all patients [2,32]. Cerebellar damage may reach a wide range, including vermis, the periphery of the anterior and posterior cerebellar lobe, the superior cerebellar peduncle, and pontine crossing tracts [2]. However, in some patients, MRI examination did not reveal any changes in the cerebellum, and it has been suspected that in these patients, destructive processes may be limited only to the vermis. Due to this fact, cerebellar atrophy may be missed in the early phase of the disease [2]. This indicates the need for regular follow-up of brain imaging tests in the group of younger patients with *COQ8A*-ataxia. On the other hand, in 25% of patients, the atrophy process spreads over the cerebellum and includes supratentorial regions, with parietal and fronto-insular cerebral atrophy and atrophy of the brainstem [2]. Global brain atrophy was also reported in patients with a severe form of the disease [32]. Typical for mitochondrial disease, stroke-like abnormalities or infratentorial signal abnormalities may also be found [2].

Among biochemical screening tests, measurement of ubiquinone in a muscle biopsy remains the gold standard test for primary coenzyme Q10 diagnosis, which enables the distinction of this group of disorders from other mitochondrial diseases [7,30,42]. It should be especially considered when cerebellar atrophy in MRI is presented [30]. The findings in muscle biopsy of COQ10D4 patients are similar to findings in other cases of primary coenzyme Q10 deficiency [7,42]. Decreased level of CoQ10 is a major finding. Moreover, biochemical analysis of respiratory-chain enzyme activities may show combined respiratory chain deficiency; reduction of complex or I + III (NADH: cytochrome c oxidoreductase) and II + III (succinate cytochrome c reductase) activity of the respiratory chain in muscle mitochondria may be detected [7,12,13,17,30,32]. Interestingly, even if the level of CoQ10 is severely decreased in muscle biopsy, the symptoms of myopathy may not be presented [13]. Although analysis of skin fibroblast may confirm these findings in muscle biopsy, normal levels of enzyme activities or CoQ10 do not exclude muscle deficiency [4,17]. Biosynthesis assays of CoQ10 biosynthesis in cultured skin fibroblasts is another test that enables confirmation of a defect in the CoQ10 biosynthetic pathway [42]. Moreover, the analysis of serum level of CoQ10 revealed that in some cases it may be within the normal range when the muscle/fibroblasts measurements showed a decreased level of CoQ10. The influence of dietary factors on the plasma concentration of CoQ10 also confirms that this parameter is not a reliable indicator of COQ10D4 [12].

Due to the heterogeneity of phenotypes and genotypes of the primary coenzyme Q10 deficiency disorders, diagnosis is established by the identification of the biallelic pathogenic variant of the *COQ8A* gene, including single-gene testing, multigene Next Generation Sequencing panel, or use of more comprehensive genomic testing, such Whole Exome Sequencing [2,15].

### 3.4. Treatment and Management

Casual treatment of *COQ8A*-ataxia remains unknown. Currently, therapy is based on the supplementation of coenzyme Q10. However, the effectiveness of the therapy differs considerably between patients [4,11,32]. It should be emphasized that approximately50% of patients may not respond to the treatment. In patients qualified as responders to CoQ10 therapy, variable results were achieved [2].

Among diverse symptoms, ataxia is found to present the greatest response to coenzyme Q10 treatment [16]. Artuch et al. reported improvement of ataxia, measured in the International Cooperative Ataxia Rating Scale (ICARS) after 3 months of treatment, and Zhang et al. observed better exercise tolerance after only 2 weeks from the therapy onset [7,33]. Moreover, total withdrawal of cerebellar signs, with the ability to walk unaided, was reported [7]. Improvement of myoclonic symptoms, tremors, dystonia, dysarthria, epilepsy, ocular disorders, mental speed, and/or muscle weakness was also described [2,7,16,17].

The optimal therapy duration has not been specified. However, the longitudinal assessment of the therapy in a small group of patients showed an improvement of −0.88 points per year in Scale for the Assessment and Rating of Ataxia (SARA) [2]. The current study revealed that the age of disease onset, disease duration at treatment initiation, ataxia severity, cumulative daily dose, mutation type, or selected clinical, imaging, or laboratory test results are not predictors for treatment response [2]. This is consistent with a previous study, where no correlation between clinical phenotype, genotype, and response to treatment was identified [16]. Contrary to these findings, Schirinzi et al. revealed a better effect of CoQ10 therapy in long-term treatment (1 year) than in patients who received medication for only 6 months. Improvement of patient outcome was represented as a decrease in the Timed 25-Foot Walk test time and SAR;, however, the study is limited due to the fact that only four patients were included [15]. Another finding that deserves emphasis is the worsening of symptoms, including ataxia and cognitive decline, after withdrawal of CoQ10 supplementation [18,33].

There are no standardized protocols for CoQ10 supplementation in COQ10D4.Based on the literature, the doses of CoQ10 range from 5 to 30 mg/kg/day or a total daily dose of 300 to 1200 mg [13,18,32,33]. Diarrhea and anorexia were found as side effects of the CoQ10 therapy. Moreover, exacerbation of myoclonus after idebenone intake was reported [32]. As in other mitochondrial disorders, the use of sodium valproate should be avoided [14].

The differences in CoQ10 dosage, duration of the therapy, heterogeneity of genotypic and phenotypic spectrum of the disease, and insufficient or contrary findings of the observational studies suggest the need for clinical trials, which will provide more detailed information and enable an individualized approach.

### 3.5. CoQ10 Pharmacokinetics

CoQ10 is a fat-soluble compound, sold as an over-the-counter oral supplement. The CoQ10 compound consists of a quinone head group responsible for antioxidant capacity and electron transfer in the mitochondrial electron transport chain and an isoprenoid side chain (tail), which provides fat solubility. The length of the isoprenoid side chain differs between species, and it is the same between humans and yeast *Schizosaccharomycespombe* [44]. Luis C. López et al. proved in the in vitro CoQ10 deficient fibroblast model that the response between short tail CoQ analogs (CoQ2) and CoQ10 differs, and they cannot be used as identical. The authors also proved that long-term supplementation of CoQ10 is necessary to improve the mitochondrial electron transport chain [45]. It is impossible to create water-soluble CoQ10 compounds without interfering with then on polar isoprenoid compound structure. However, multimolecular spatial structure modifications can be performed, as described in Section 3.7.

Available CoQ10 supplements are mostly produced in yeast fermentation [46]. The end products of fermentation are polymorphic crystal structures that need to be dissociated into a single CoQ10 molecule, the only bioavailable structure. In the study of López-Lluch G., the authors determine factors that affect bioavailability. Bioavailability was quantified as the area under the curve (AUC) at 48 h and as peak plasma CoQ10 concentration (Cmax) after the intake of 100 mg CoQ10 capsules. There are two forms of CoQ10 currently on the market: the oxidized form (ubiquinone) or the reduced form (ubiquinol). Regardless of the initial orally taken form, CoQ10 is transported in the bloodstream as the ubiquinol form in conjunction with low-density-lipoproteins and very-low-density-lipoproteins. In the study, bioavailability of the crystalized ubiquinol form was greater than the bioavailability of the crystalized ubiquinone form (AUC and Cmax values were 14.8 mg/L/48 h and 0.49 mg/L for ubiquinol vs. 6.89 mg/L/48 h and 0.33 mg/L for ubiquinone, respectively). Compounds used in the study differed in the preservatives, such as vitamin C. The authors suggested that the difference in better bioavailability of the ubiquinol depends on the type of antioxidant preservatives and not on the redox form of CoQ10 [47].

According to the López-Lluch G. study, the most important factors affecting bioavailability are preservatives and individually unknown physiological factors. A significant role in bioavailability showed oil composition in favor of soyabean oil versus matrices such as olive oil and cocoa butter, or olive oil and soy oil [47]. The gelatine capsule material dissolves in the stomach within minutes and seems to have little importance in CoQ10 bioavailability. The stomach transit time depends on the type of recently eaten food and varies among individuals [48]. In the duodenum, bile from the gallbladder interacts with CoQ10 to form a micellar structure with a diameter up to 20nm. Inside micelles, there are thousands of CoQ10 molecules. Micelles form and break apart easily and constantly. After contact with the surface of the enterocytes, only one CoQ10 molecule is absorbed in a facilitated diffusion process without a micellar envelope [48]. Many studies have shown an increase in plasma CoQ10 values as the dose increased. In contrast, the increase per orally taken 100 mg CoQ10 tended to decrease [49,50,51]. This information leads us to suspect the existence of a transporter in the enterocyte wall. So far, only cholesterol Niemann-Pick C1 like transporter is linked to CoQ10 absorption [52]. CoQ10 exits the enterocytes within the chylomicrons in the process of exocytosis and then is transported with chyle within lymphatic vessels to reach the systemic circulation. In the liver, CoQ10 is linked to low-density lipoproteins or very low-density lipoproteins. In addition, CoQ10 is carried in the platelets and leukocytes, and only a small amount of CoQ10 is in the erythrocytes. Typically CoQ10 reaches the highest plasma concentration after 6 h, and the half-life of the absorbed CoQ10 is approximately 33 h. So far, there is no discovered transporter from the lipoprotein carriers in the blood to all cells [48].

It is assumed that the transport of nonpolar structure CoQ10 relies on simple diffusion. According to this statement, in the primary CoQ10 deficiency diseases, serum CoQ10 concentration is higher than in the affected tissues [48]. Matthews et al. and Smith et al. proved that in orally supplemented rat and mice brain, CoQ10 concentration increased [53,54]. According to animal studies, CoQ10 can cross the blood–brain barrier (BBB).

### 3.6. Outcomes

*COQ8A*-ataxia is a progressive condition with relatively mild to moderate overall disease progression [2]. Compared to other childhood-onset ataxias, the symptoms of the ataxia are usually mild to moderate, depending on the functional assessment scores, ranging from 1.5 to 7 (mean SDFS 2.8) [2,32]. According to a recent study, the median ataxia progression rate was calculated at 0.47 SARA points per year [2]. In another study, SARA after long-term observation ranged from 4 to 15.5/40 (mean 10.7, standard deviation 3.8) [32]. No correlation between the age of ataxia onset and duration of ataxia was found. Moreover, children with early onset of the disease are reported to have a usually mild or moderate disease course, even though cases presented as infantile encephalopathy were reported. The disability range was thought to be mild, since most patients were still ambulatory after a long period of ataxia history (medium of 20.8 years) [32]. However, a recent study suggests that in more than 50% of patients it leads to dependence on a walking aid or wheelchair [2]. It should be pointed out that there is also a group of children who never learned to walk or are severely intellectually disabled [32]. Moreover, severe courses of the diseases, with premature death, status epilepticus, spastic tetraparesis, and severe dilated cardiomyopathy, have been reported [12,32].

### 3.7. Approach to Further Therapies

Improvement of bioavailability may be particularly crucial for patients with primary coenzyme Q10 deficiency. It has been suggested that the bioavailability of CoQ10 may be severely limited due to its water insolubility and instability. To evaluate if improvement in the absorption and bioavailability of CoQ10 may have a beneficial effect on primary coenzyme Q10 deficiency, García-Corzo et al. compare the effect of ubiquinone-10 and its reduced form, ubiquinol-10 (a form of ubiquinol, which is obtained by reduction of the CoQ10), in water-soluble formulations in a mouse model of (Coq9X/X) of mitochondrial encephalopathy. The study proved that oral ubiquinol-10 supplementation resulted in a greater increase of CoQ10 in all tissue, including the cerebrum and cerebellum, compared to ubiquinone-10. However, only ubiquinol-10 led to an increase in the levels of CoQ10 in mitochondria from the cerebrum in mice. Moreover, animals treated with ubiquinol-10 presented increasing body weight and CoQ-dependent respiratory chain complex activities. Reducing the vacuolization, astrogliosis, and oxidative damage in the diencephalon, septum–striatum, and, to a lesser extent, in the brainstem was also described [55].These findings suggest that due to better bioavailability, tissue distribution, and ameliorating of the encephalopathy phenotype, ubiquinol-10 may be beneficial also for patients with COQ10D4; the effect of this therapy should be verified in a model of *COQ8A*-ataxia.

Recently, other approaches to improve COQ10 bioavailability have been proposed. Cui et al. developed a water-soluble coenzyme Q10 micelle and proved that plasma CoQ10 level was higher after administration of water-soluble CoQ10micelle than lipid-soluble CoQ10 in a rat model of chronic tacrolimus nephropathy [56]. Zhang et al. revealed that a CoQ10-loaded nanoemulsion based on PNO (Pinuskoraiensis nut oil) has better bioavailability and absorption than standard CoQ10 in amodel of mouse colon cancer cells [57]. Another study confirmed this observation, as it proved that whey protein nanoparticles loaded with coenzyme Q10 improved the in vitro antioxidant activity of CoQ10 and its bioaccessibility during simulated gastrointestinal digestion [58].

Moreover, Masotta et al. suggested that the oleogel matrix, which can support a high dose of oil-dissolved CoQ10, may be beneficial for patients with CoQ10 deficiency who required high doses of CoQ10. The advantages are higher CoQ10 increase in plasma in repeated dose study, higher apparent plasma half-life, and also, clinically, oleogel-loaded-CoQ10 is easy to swallow by patients who suffer from secondary dysphagia, which may contribute to better adherence to therapy [59].

Another point that should be discussed is that coenzyme Q10 brain distribution as improvement of neurological symptoms, which are the main features of COQ10D4, is rarely achieved. A potential challenge of CoQ10 therapy in neurological disorders is its limited BBB penetration. Wainwright et al. revealed lipoprotein-associated transport of CoQ10 via BBB in the uptake of CoQ10, SR-B1 (Scavenger Receptor), and RAGE (Receptor for Advanced Glycation End products); the efflux proceeds via LDLR (Low Density Lipoprotein Receptor) transporters, which results in no “net” transport of CoQ10 via BBB; thus, the accumulation of CoQ10 in the brain is not achieved because of opposing transport systems. However, in a mouse model of CoQ10 Deficient Endothelial Cell Culture, it was indicated that BBB tight junctions were disrupted and CoQ10 “net” transport to the brain side was increased. Moreover, the intake of CoQ10 was not improved by the addition of other antioxidants. These findings suggested that in primary coenzyme Q10 deficiency, CoQ10 distribution in the brain may be improved by the administration of LDLR inhibitors or by interventions to stimulate the luminal activity of SR-B1 transporters [60]. Better CoQ10 brain distribution and peak plasma concentration were achieved in rat models using a self-microemulsifying drug delivery system of CoQ10 compared to the coenzyme Q10 suspension [61]. Sheykhhasan et al. reported a positive effect of CoQ10 drug delivery with the use of exosomes derived from adipose-derived stem cells (ADSCs-Exo) in a rat model of Alzheimer’s disease [62].

Further studies are required to investigate whether water-soluble CoQ10, CoQ10 loaded with nanoparticles or nanoemulsions, or self-emulsifying systems may have beneficial effects in COQ10D4 models.

Due to inconsistent responses to oral ubiquinone-10 supplementation among patients with COQ8A-ataxia, CoQ10 analogs may be considered as a potential therapy. Among various ubiquinone analogs, attempts to administer idebenone to patients with COQD4 have been described [32,63]. Idebenone is a synthetic quinone, which presents similarity to natural CoQ10; however, it differs structurally by the presence of a much shorter and less lipophilic tail. This contributes to pharmacokinetic changes, which differentiate idebenone from CoQ10, such as much faster intestinal absorption and metabolization within minutes of its administration, which leads to no detection in plasma after 1 h. Similarly to CoQ10,Idebenone acts as an efficient substrate for mitochondrial complex II and III; however, contrary to CoQ10, it may inhibit mitochondrial complex I. Nevertheless, it has been proven that idebenone bypasses complex I and activates an alternative pathway with cytoplasmic enzyme NADH-quinone oxidoreductase, which reduces Idebenone. Then, it enters mitochondria, where it is directly oxidized by complex III. Although the idebenone activation of mitochondrial respiratory complexes is not required, it has been shown that the physiological level of CoQ10 is necessary for the activation of the alternative glycerophosphate pathway of idebenone [64], which may limit its use in COQ10D4. Moreover, clinical trials proved the safety and efficacy of higher doses of Idebenone in conditions such as Parkinson’s disease, LHON, or MELAS syndrome [64]. In rat models of BBB in CoQ10 deficiency, idebenone presented an intake to the brain greater than efflux. Explained by the direct BBB transport rather than as a part of lipoprotein. This possibly better than CoQ10 brain distribution indicated that it may have a better clinical outcome in COQ10D4. However, these potential benefits of the idebenone therapy have not been reflected in clinical practice, as patient reports revealed poor outcomes of idebenone treatment in CoQ10D4.Mignot et al. reported aggravation of myoclonus in one patient after idebenone administration and no effect in another patient [32]. Mollet et al. and Aure et al. also observed a worsening in the patient’s condition after switching from CoQ10 to idebenone [5,63]. It should be highlighted that these data are limited due to a small number of documented idebenone administrations. However, a study on fibroblasts of patients with CoQ10 deficiency revealed a decreased level of CoQ10 in cells after idebenone, and no increase in ATP level, although increased superoxide anion production and oxidative stress-induced cell death were detected [45]. These findings lead to the conclusion that COQ10D4 should be treated with CoQ10, not idebenone.

Interestingly, CoQ4, another coenzyme Q10 analog with a short isoprenoid side chain, which results in less lipolytic properties, may effectively replace CoQ10 in the therapy of coenzyme Q10 deficiency [64,65]. Although this group of CoQ10 analogs is found to have toxic characteristics, CoQ4 displays only minimal toxicity [64,66]. Dietary supplementation with CoQ4 was found to successfully rescue neural phenotypes in the Drosophila model of coenzyme Q10 deficiency [66]. Cerqua et al. found that coenzyme Q4 supplementation leads to restoration of normal mitochondrial function CII + III activity in human fibroblasts with *COQ2* mutation and human cell lines harboring a genetic ablation of *COQ4*, as well as increasing ATP levels. However, after CoQ4 administration, the CoQ10 level in cells remained unchanged [65].

Other CoQ10 analogs, such as mitoquinone, decylubiquinone, vatiquinoneplastoquinone, and SKQ1, have been reported to have a beneficial effect on various disorders related to CoQ10; many of them presented with additional attributes, like better bioavailability [64]. As the use of these agents has not been described in primary coenzyme Q10 deficiency models, further studies are obligatory to define their usefulness in this particular group of disorders.

Analogs of 4-hydroxybenzoic acid (4-HB), which are benzoquinone ring precursors for the synthesis of CoQ, have been proposed as another approach to therapy in coenzyme Q10 deficiency. 4-HBA was found to fully restore endogenous CoQ10 biosynthesis in *COQ2*-deficient cell lines with increased expression of *COQ4* and *COQ7* protein and improve cell viability. 4-HBA acts as a direct substrate of COQ2, which mediates its condensation with the poly is oprenoid side chain [67]. Lopez et al. revealed that vanilla acid in a human cell line lacking functional *COQ6* contributes to recovering CoQ biosynthesis and ATP production, as well as restoring ROS production to a normal level [68]. Moreover, vanillic acid supplementation was found to increase the production of CoQ10 in cells with *COQ9* deficiency with a dose-dependent effect and to improve cell viability [69]. β-Resorcylic Acid (2,4-dihydroxybenzoic acid) contributes to increasing cellular CoQ10 levels, as it specifically bypasses the *COQ7* deficiency [69,70]. It has been shown that supplementation of 2,4-dihydroxybenzoic acid in mouse models of *COQ9*-deficient cells also resulted in increased CoQ10 levels; however, the effect was dependent on the specific mutation as a result of different levels of COQ biosynthetic proteins [71]. It should be pointed out that *COQ8A* acts as a kinase that phosphorylates other COQ proteins and stabilizes the Q protein complex; it is not directly related to 4-HBA and other substrates, which differentiates this mechanism of action from other models of CoQ10 deficiency. Thus, these solutions may not be efficient for patients with COQ10D4.

## 4. Differential Diagnosis of Other Mitochondrial Disorders Presenting with Ataxia

Cerebellar ataxia is the most common presentation of primary coenzyme Q10 deficiency syndromes and is also a predominant clinical feature of COQ10D4, which may distinguish this condition from other diseases in this group [2,4]. However, it should be highlighted that patients with COQ10D4 may present multiple additional neurological and non-neurological symptoms. Moreover, the spectrum of symptoms of encephalopathy, intellectual disability, seizures, and muscle involvement has been described in patients with variants responsible for other primary coenzyme Q10 deficiency (*COQ2*, *COQ4*, *COQ6*, *COQ7*, *COQ9*) as well as in patients with *COQ8A*-ataxia. In the case of patients who do not present the ataxia simplex phenotype, and due to similarity in laboratory findings and a wide spectrum of overlapping of other neurological symptoms, it may be hard to differentiate among primary coenzyme Q10 deficiency without genetic testing [4,72].

Apart from the group of primary coenzyme Q10 deficiency, other primary mitochondrial diseases should be taken into account during differential diagnosis, as ataxia is one of the main features of CNS dysfunction in this group of disorders.

Leigh syndrome (LS) is the most common mitochondrial disease in the pediatric population with a heterogeneous clinical phenotype. Most patients present first symptoms before the age of 2 years; however, late-onset Leigh syndrome has also been frequently reported [73]. The classical clinical features include psychomotor delay or regression of development, muscle weakness, truncal ataxia, seizures and intention tremor, and respiratory dysfunction [73,74]. Moreover, for patients with the late-onset form of the disease, ataxia and motor weakness are characteristic of the first symptoms [73]. As in other mitochondrial diseases, lactic acidosis of the blood or CSF is observed. Focal, necrotizing lesions, typically of the basal ganglia, diencephalon, or brainstem, are described as hallmarks of the LS; however, the occurrence of cerebellar atrophy may also be frequently presented [73,75]. LS is associated with genotypic heterogeneity, and it should be emphasized that in many genes related to the condition, *PDSS2* gene, which is related to primary coenzyme Q10 deficiency, has also been described [74].

Another mitochondrial disease that may present with ataxia is Kearns-Sayre syndrome (KSS). Most cases of the disease are sporadic and are caused by the large deletion of mitochondrial DNA, which leads to dysfunction in oxidative phosphorylation. KSS belongs to the group of chronic progressive external ophthalmoplegia(CPEO) disorders. Typically, patients present with ptosis, followed by progressive ophthalmoplegia and pigmentary retinopathy. Apart from cerebellar ataxia, other neurological features include nystagmus, hearing loss, and dementia. Among various features, complete heart block, cerebrospinal fluid protein greater than 100 mg/dL, renal dysfunction, endocrinologic abnormalities including short stature, and diabetes mellitus are significant findings. Affected patients develop their first symptoms before the age of 20 [76,77].

Myoclonus, dementia, myopathy, seizures, and cerebellar ataxia are classical features of MERRF (myoclonus epilepsy associated with ragged-red fibers) syndrome. Other features such as lipoma, diabetes mellitus, optic atrophy, peripheral neuropathy, hearing loss, and dementia have also been reported [78,79]. The course of the disease may be diverse even within variable phenotypes in the same family. MERRF typically presents as juvenile or early-adult onset, with progressive myoclonus epilepsy. Significant histopathological findings are ragged red fibers in muscle tissue and an MRI that may show cerebellar and brainstem atrophy [78,80]. Maternally inherited mutations in the *MTTK* gene, encoding tRNA Lys, are detected in most cases [78,79]. Moreover, overlapping genotypes with MELAS have also been described.

Myoclonus Epilepsy Myopathy Sensory Ataxia (MEMSA) is a condition that belongs to polymerase gamma(*POLG*)-related disorders. It is characteristic of MEMSA that apart from the occurrence of myopathy, epilepsy, and ataxia, ophthalmoplegia does not appear. The onset of the disease is typically related to sensory polyneuropathy, which may lead to ataxia. The lack of the ragged red fibers in muscle biopsy distinguishes this condition from MERRF [81]. Another feature of *POLG*-related disorders is Ataxia Neuropathy Spectrum (ANS), where predominant symptoms like neuropathy and ataxia are accompanied by mild cognitive impairment, involuntary movements, psychiatric symptoms, myoclonus, blindness, hearing loss, and liver involvement or ophthalmoplegia without the occurrence of myopathy [81].

Neuropathy, ataxia, and retinitis pigmentosa (NARP) are caused by a mutation in the gene encoding subunit 6 of mitochondrial H(+)-ATPase (*MTATP6*). Similar to COQ10D4, the clinical presentation includes developmental delay, seizures, ataxia, and muscle weakness. Moreover, rarely described in COQ10D4 patients: retinitis pigmentosa, dementia, and sensory neuropathy [82,83]. The age of presentation is variable; however, in this condition symptoms of ataxia are usually revealed in the older age [84].

Sensory ataxic neuropathy, dysarthria, and ophthalmoparesis (SANDO) are other autosomal recessive mitochondria that may be the cause of the ataxia. Nevertheless, it is usually characterized by adult-onset sensory ataxia (gait ataxia), dysarthria, seizures, myopathy, and hearing loss, which show in the adult age [85].

## 5. Conclusions

In this article, we provide a detailed description of a patient with *COQ8A* mutation, broadening the phenotypic spectrum of COQ10D4. It should be emphasized that the described patient had already presented symptoms of cerebellar ataxia, but first and control MRI examinations did not reveal signs of cerebellar atrophy—typical features of COQ10D4.This was contrary to findings described by Mollet et al., which revealed that brain imaging demonstrates cerebellar atrophy in all patients by the time cerebellar signs have already appeared [5]. Patients with *COQ8A*-ataxia may present variable symptoms, appearing not only in childhood; thus, the depicted spectrum of mitochondrial diseases may overlap with the COQ10D4 phenotype, and genetic testing may be the only tool to provide an accurate diagnosis. Moreover, in the group of coenzyme Q10 deficiency, it can replace the invasive test of muscle biopsy. Despite a large number of published articles, current knowledge doesn’t answer the question of why CoQ10 supplementation does not work in all patients. Clinical trials are needed to evaluate the effectiveness of the therapy and the influencing factors. The supplement with antioxidant preservatives should be taken several times a day with a meal. The effectiveness of the therapy should be assessed after achieving stable CoQ10 concentration, by taking blood samples and measuring the concentration of the CoQ10 in leukocytes or platelets. Future studies should focus on searching for transporters associated with the intake of CoQ10 from the gastrointestinal tract and the ability to cross the blood–brain barrier. There are several proposed alternative therapies for primary coenzyme Q10 deficiency, such as water-soluble formulations of CoQ10, LDLR inhibitors, or new analogues of CoQ10. Making references to *COQ8A*-ataxia patients is severely limited due to the lack of studies conducted on models of COQ10D4.
